# Supplementary material for: Feature-based reward learning shapes human social learning strategies
Source: Nat Hum Behav. 2025 Jul 23;9(10):2183–98. doi: 10.1038/s41562-025-02269-4 (PMC12545201; doi:10.1038/s41562-025-02269-4)
Supplement: Supplementary file 1 — Supplementary Figs. 1–15, Table 1, Sections 1–4 and References. [file 41562_2025_2269_MOESM1_ESM.pdf]

nature human behaviour

Supplementary information

https://doi.org/10.1038/s41562-025-02269-4

Feature-based reward learning shapes human social learning strategies

In the format provided by the authors and unedited

Table of Contents

Figure S1 2
Figure S2 3
Figure S3 5
Figure S4 6
Figure S5 7
Figure S6 8
Figure S7 9
Figure S8 10
Figure S9 11
Figure S10 12
Figure S11 13
Figure S12 14
Figure S13 15
Figure S14 16
Figure S15 17

Table S1 18

Section 1: Computational Models 19
1.1 Fixed Heuristics 19
1.2 Value Shaping 20

Section 2: Additional Analyses 21
2.1 Entire Test Phase 21
2.2 Social Network Index 21
2.3 Overlearning 22
2.4 BEAST Task Robustness Test 22
2.5 Order Effects 22

Section 3: Model Identifiability and Generalizability 23
3.1 Model Recovery 23
3.2 Generalizability 25

Section 4: Additional Pre-registered Analyses 25
4.1 Exclusion Criteria 25
4.2 Terminology 26
4.3 Feature competition predictions 26

Supplementary References 27

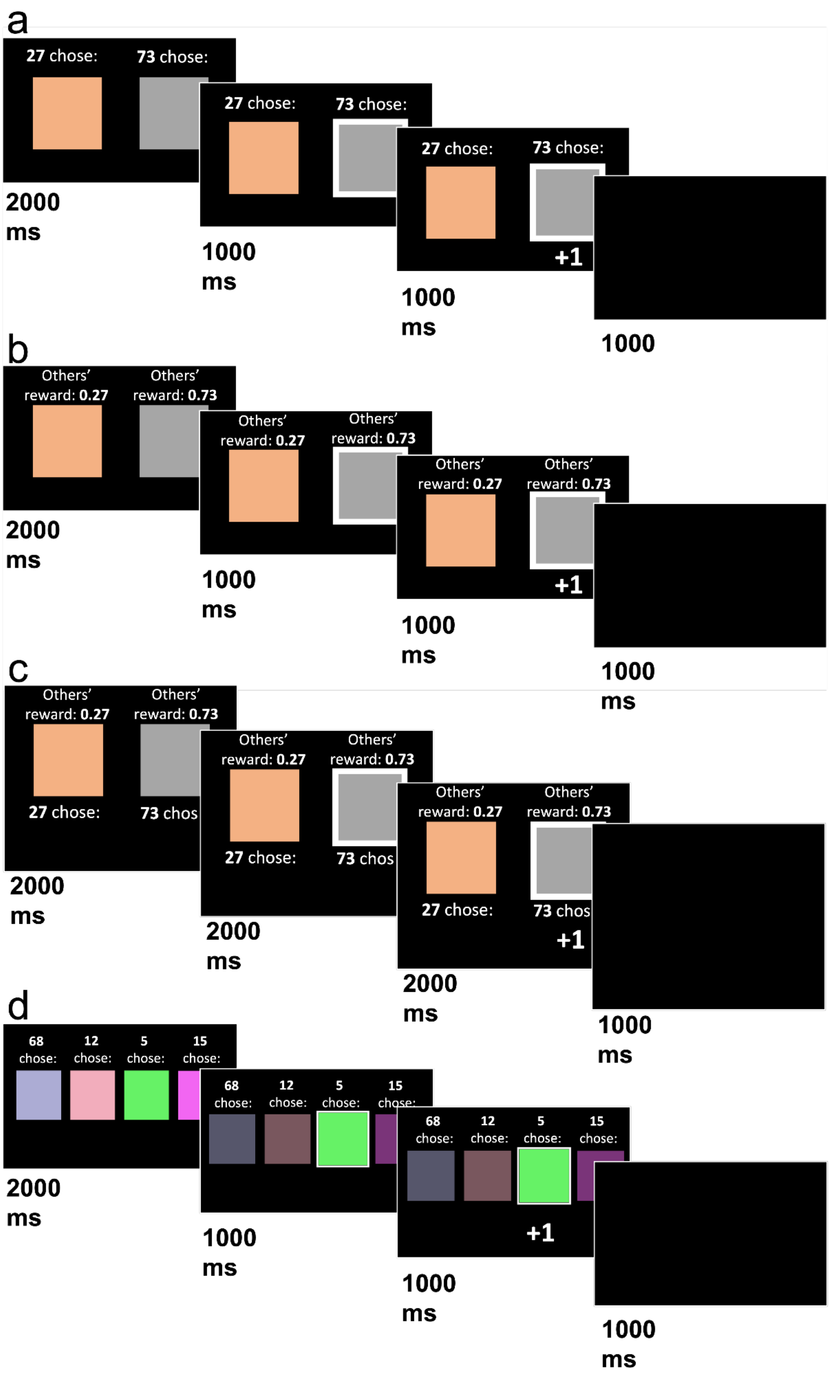

Figure S1. Overview of a trial of the learning phase in the experimental task. (a) The task used in Experiments 1, 3 & 5 with others' choices as the social feature. (b) The task used in Experiment 2, with others' payoff as the social feature. The frame indicates the option selected by the participant. (c) Task design of Experiment 3. A combination of the two social features 'others' choices' and 'others' payoffs' are visible in each trial (position randomized between participants). (d) Task design of Experiment 4. Four static options were available during the Learning phase, alongside the social feature 'others' choices'. The Test phase featured four novel options. The task design of Experiment 6 is depicted in Figure 4 of the main text.

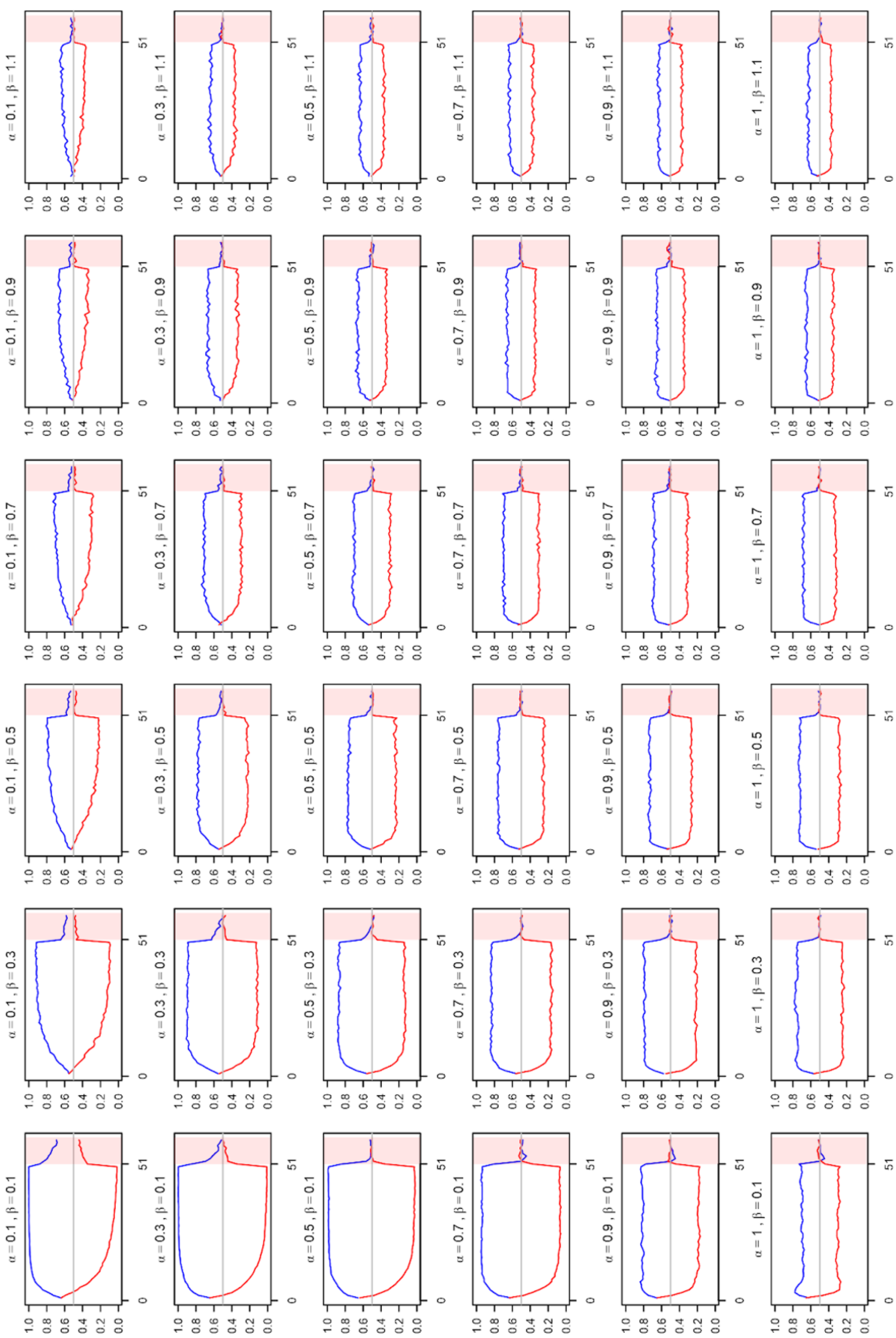

Figure S2. Apriori SFL model predictions for Experiment 1, across a range of α and β parameter values. The weight prior parameter was kept constant at 0.1. Blue lines indicate the Congruent condition and red lines the Incongruent condition. The shaded red area indicates the Test phase.

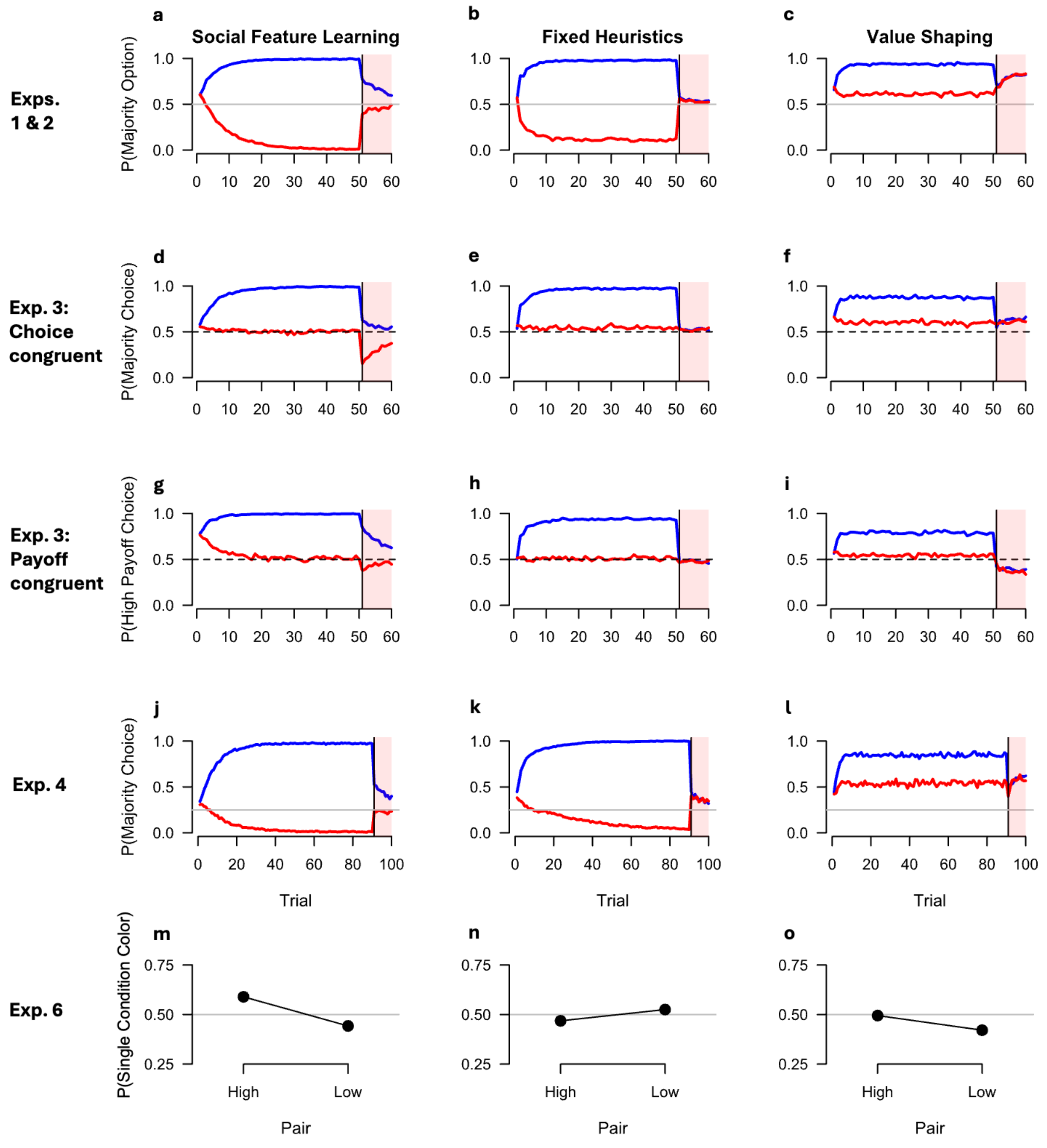

Figure S3. Out-of-sample model simulations for all experiments. For all experiments, models were simulated based on the median estimated parameters from Experiment 1. (a-c) Experiment 2 (c.f. Figure 2, main text). Note that the models make the same predictions for Experiments 1 & 2 (and the Learning phase of Experiment 5). (d-i) Model simulation of Experiment 3 (c.f., Figure 3, main text), displayed separately for the Choice congruent and the Payoff congruent condition. (j-l) Model simulation of Experiment 4 (c.f., Figure 3, main text). (m-o) Model simulation of the Experiment 6 Transfer phase, showing the predicted probability of choosing the single condition target for the high and low reward value pairs (c.f., Figure 4, main text). The SFL and Value Shaping models both include prior parameters. For the payoff feature in Experiment 4, the estimated prior parameters from Experiment 2 were used. Blue lines indicate the Congruent condition and red lines the Incongruent condition. The shaded red area in panels c, f, i & l denotes the Test phase, where new choice options were introduced.

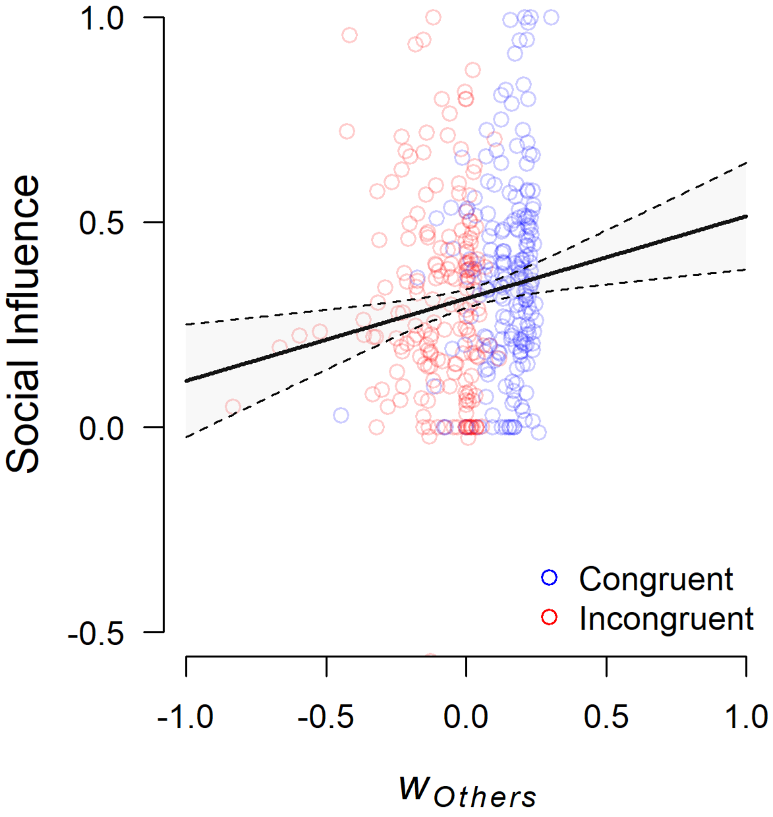

Figure S4. Estimated social feature weights predict social influence in Experiment 5. The individually estimated social feature weight, w_{Others}, from the last trial of the Learning phase predicted susceptibility to social influence, S_{Beast}, in the BEAST task. See main text for statistics and "*Section 2: Additional Analyses*" for robustness tests. Confidence bands indicate 95% CI.

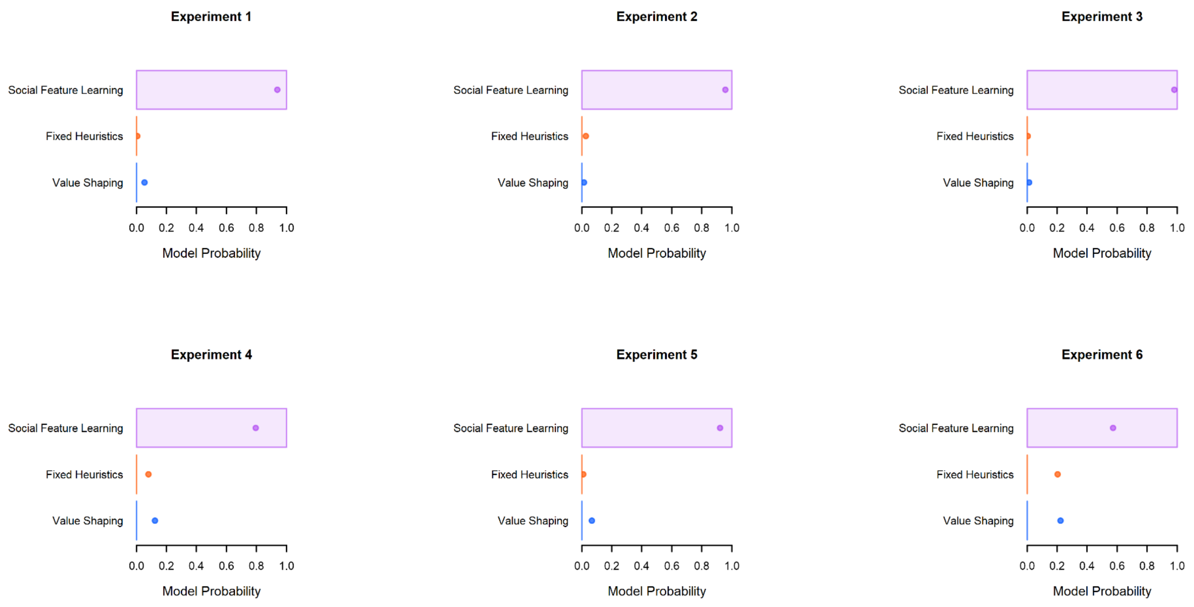

Figure S5. Model comparison for individual experiments. The figures show, for each of the experiments, the model probability (protected exceedance probability) of each model, representing the probability that this model is the most common in the population. The dots indicate the posterior model frequencies, the estimated prevalence of each model in the participant population. The results demonstrate that the SFL model provided the best account of the data from all experiments.

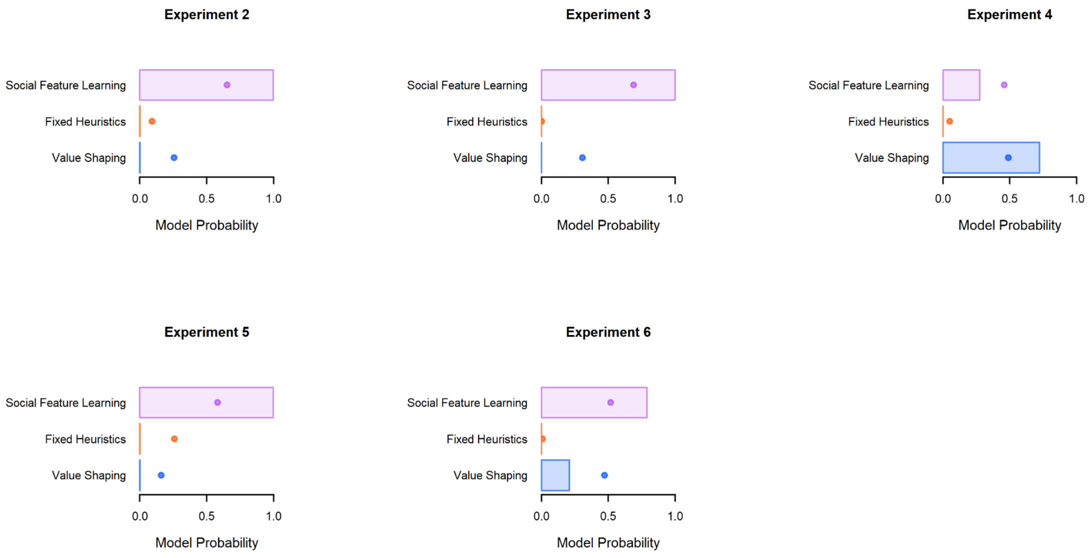

Figure S6. Out-of-sample predictions. Model probability (bars) and posterior frequencies (dots) for Experiments 2-6, based on the median estimated parameter values from Experiment 1.

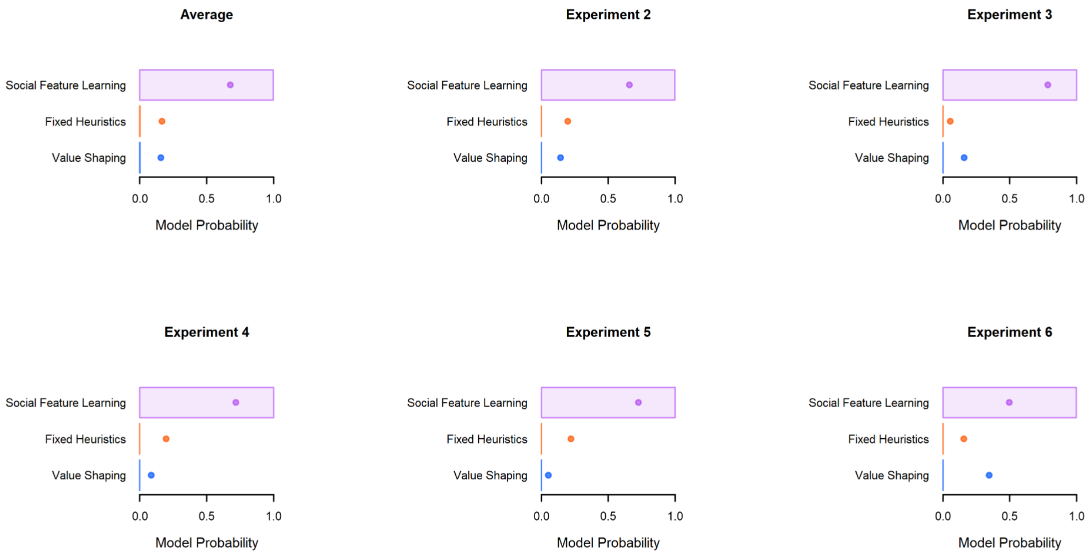

Figure S7. Out-of-sample predictions. Model probability (bars) and posterior frequencies (dots) for Experiments 2-6, based on the mean estimated parameter values from Experiment 1. The top left panel depicts the average.

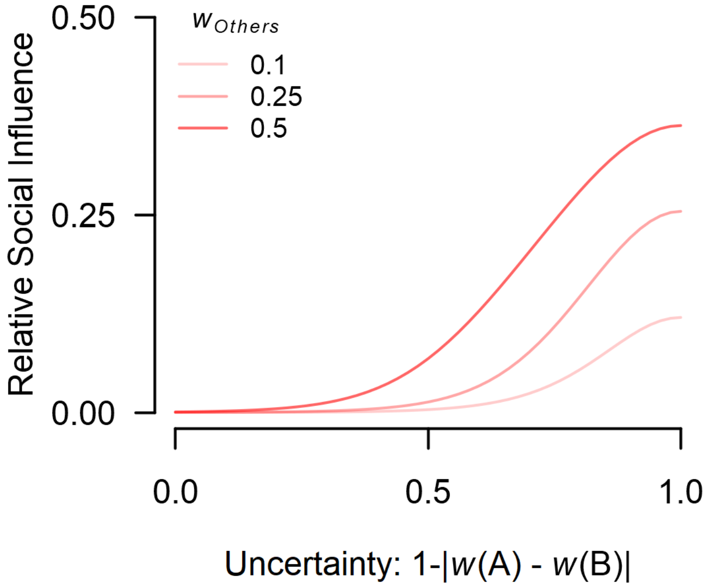

Figure S8. Uncertainty increases relative social influence. The figure illustrates how relative social influence, defined as the probability of selecting an option in the presence versus absence of others, changes as a function of uncertainty, for three levels of w_{Others}. Social influence is highest when uncertainty is maximal.

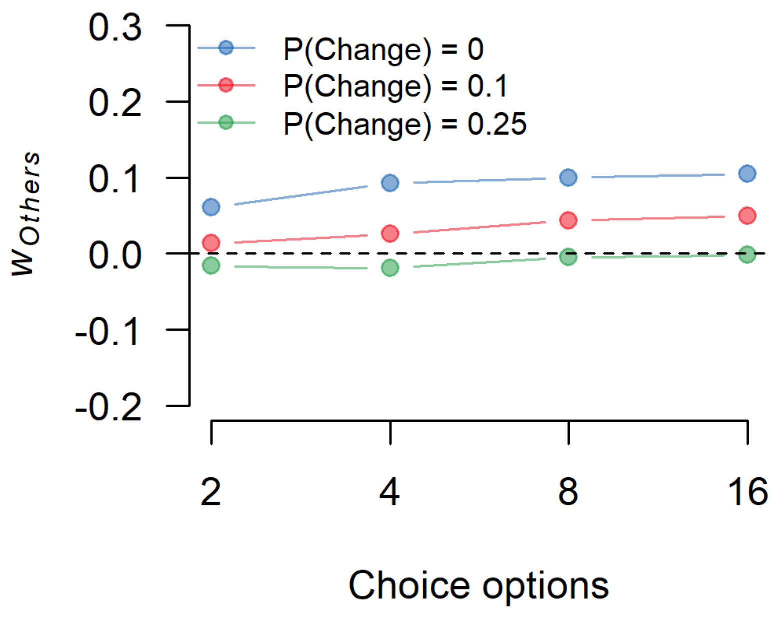

Figure S9. Multiple choice options in the agent-based simulations. The figure displays the median value of W_{Others} across 800 simulation runs for each combination. The number of simulation time steps was proportional to the number of options (2 options = 100 time steps, 4 options = 200 time steps, 8 options = 400 time steps. For 16 options, we increased to 1600 time steps, since preliminary simulations based on 800 time steps showed that the average feature weight was still growing at the end of the run).

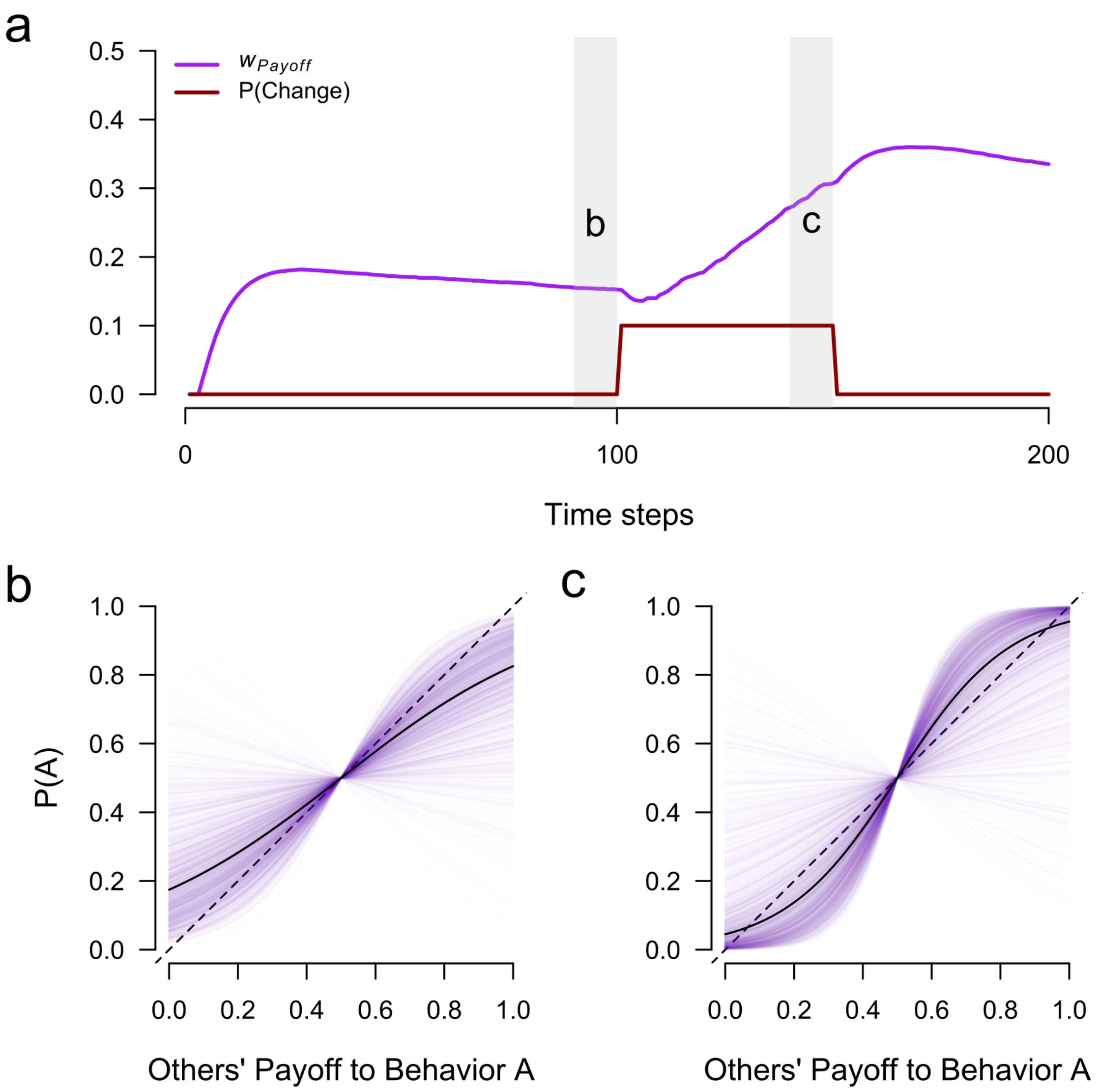

Figure S10. Within- and between-individual variability in payoff-biased social learning. The figure illustrates how environmental unpredictability impacts payoff-biased learning (w_{Payoff}) according to the SFL model. The weight of others' payoffs increases when the environment is more unpredictable (a). The grey vertical bars indicate the time points depicted in b-c. (b-c) Social influence functions. When making decisions between unknown options the same (500, random) individuals exhibit less (b) or more (c) reliance on the payoff feature, depending on the current state of the environment in (a) The solid black lines illustrate the average behaviour, while the dotted horizontal line indicates random copying

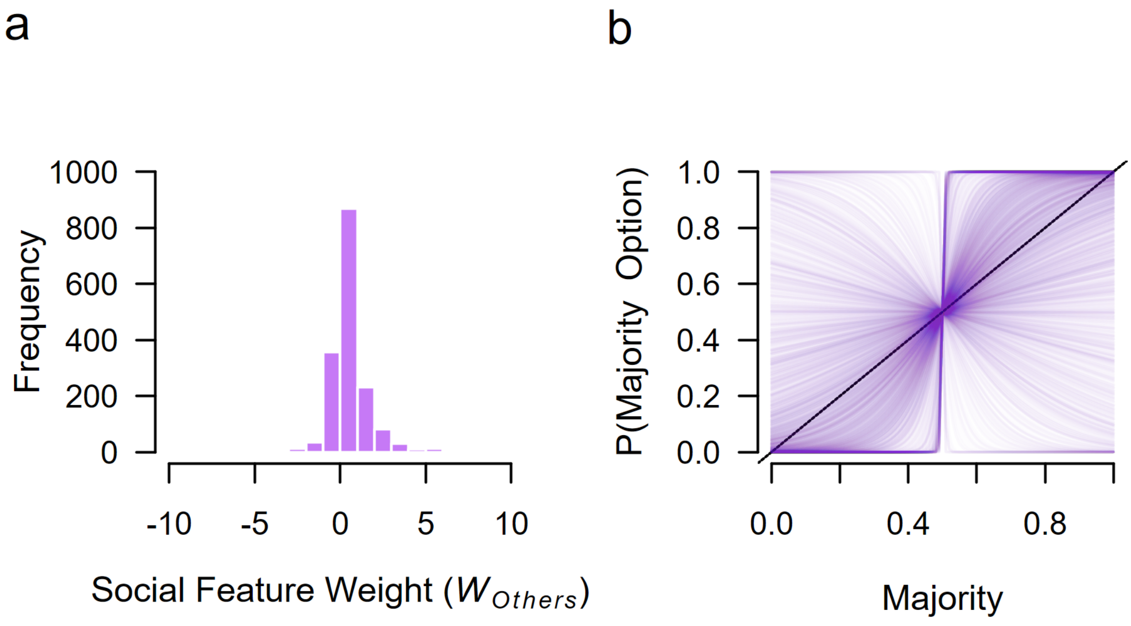

Figure S11. Between-individual variation in majority-biased social learning. (a) Variation in the estimated w_{Others} prior from Experiments 1 & 3-6, using the SFL+prior model. Participants with absolute w_{Others} values exceeding 10 were excluded for display purposes. (b). Inferred social influence functions based on estimated SFL+prior parameters. Each line represents one participant from Experiments 1 & 3-6. These results demonstrate a large pre-experimental variability in the weight of other's actions, consistent with varying learning histories.

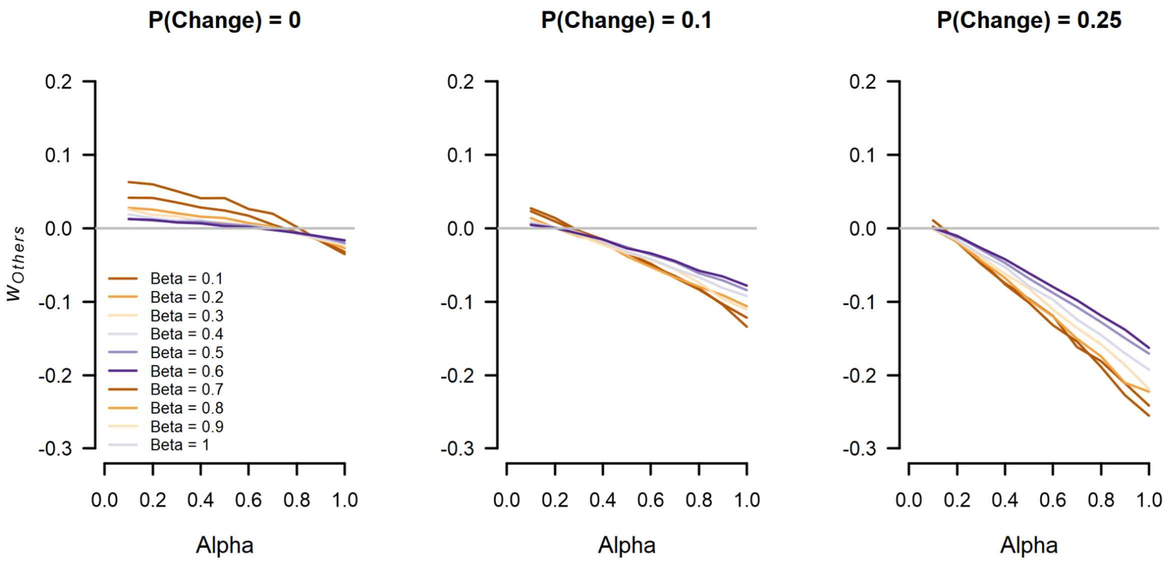

Figure S12. The median simulated social feature weight $W_{Others,}$ as a function of different values of the SFL parameters alpha (α) and beta (β). Each value represents the median of 800 simulation runs. For all other analyses, alpha = 0.2 & beta = 0.1

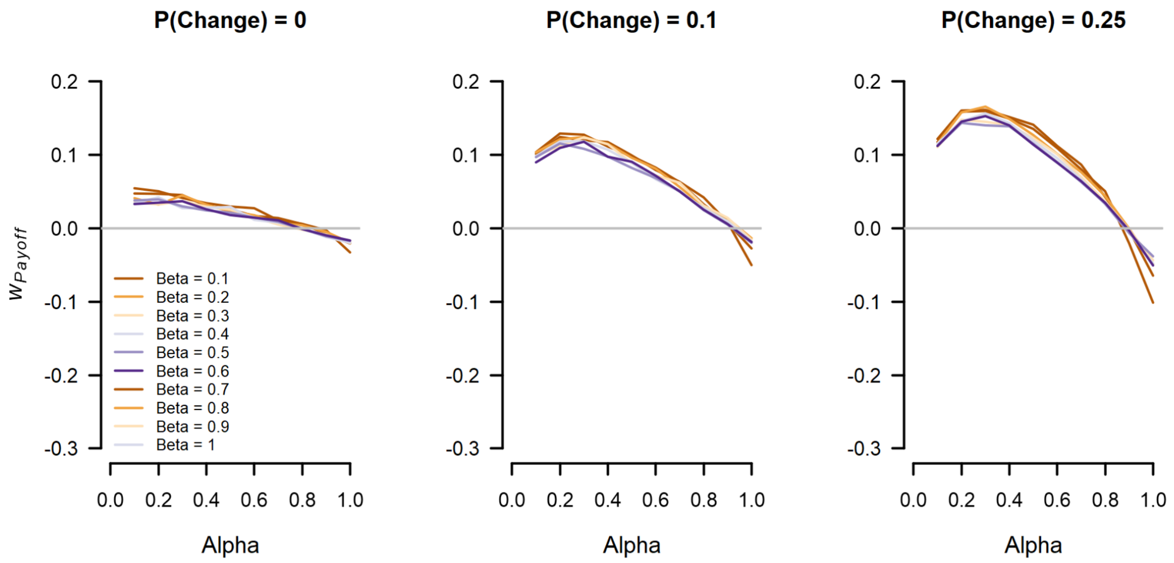

Figure S13. The median simulated social feature weight $W_{Payoff,}$ as a function of different values of the SFL parameters alpha (α) and beta (β). Each value represents the median of 800 simulation runs. For all other analyses, alpha = 0.2 & beta = 0.1

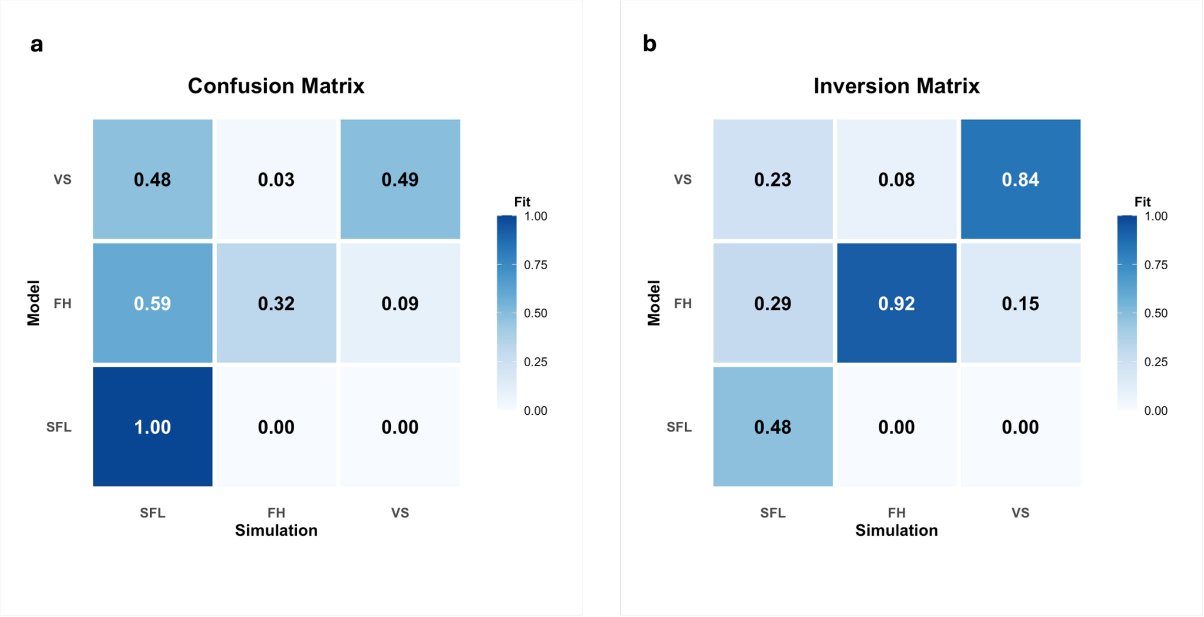

Figure S14. Confusion (a) and inversion (b) matrices for the SFL model without a prior parameter. The analysis was based on simulations of Experiment 1.

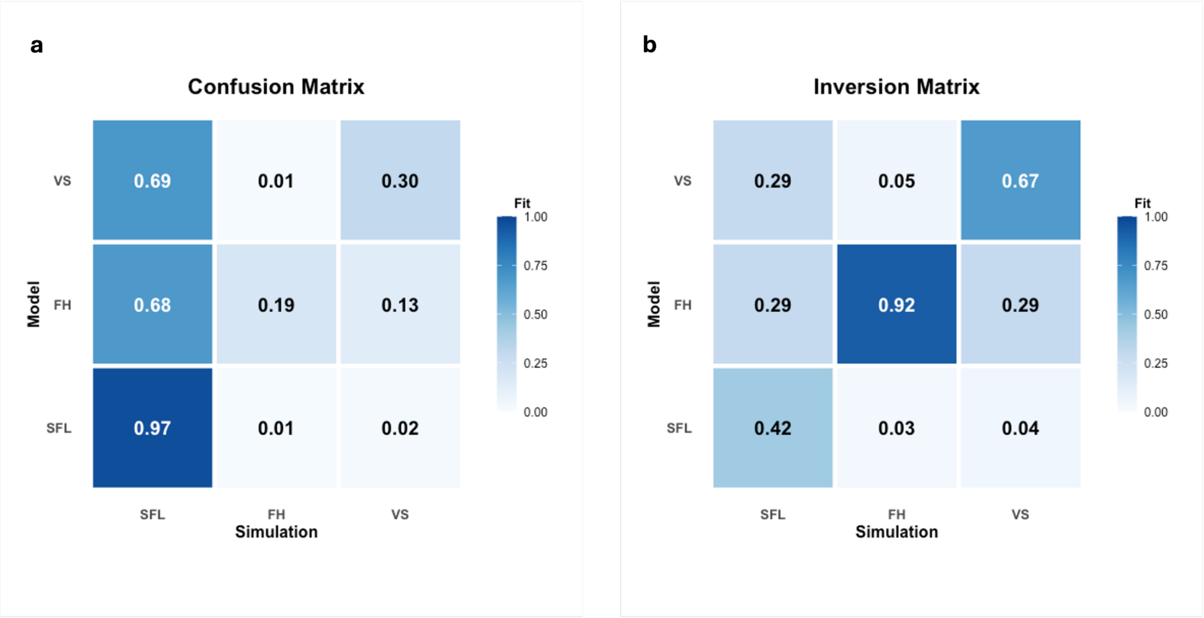

Figure S15. Confusion (a) and inversion (b) matrices for the SFL model with a prior parameter. The analysis was based on simulations of Experiment 1.

Exp.	Parameter	Min	25%	Median	75%	Max
1	α	0	0.043	0.15	0.363	1
1	β	0.01	0.016	0.146	0.285	1
1	P	-2.016	-0.007	0.117	0.614	12.387
2	α	0	0.048	0.158	0.37	1
2	β	0.01	0.019	0.127	0.278	1
2	P	-2.638	0.066	0.322	1.183	15.749
3	α	0	0.003	0.109	0.472	1
3	β	0.01	0.07	0.306	0.617	1
3	P_{choice}	-18.702	0.072	0.667	1.503	33.856
3	P_{payoff}	-5.804	0.221	1.188	2.171	35.773
4	α	0	0.081	0.208	0.374	1
4	β	0.01	0.102	0.17	0.258	1
4	P	-2.056	-0.008	0.237	0.667	20.137
5	α	0	0.027	0.222	0.475	1
5	β	0.01	0.01	0.109	0.264	1
5	P	-7.603	0.003	0.2	1.127	95.965
6	α	0	0.051	0.229	0.482	1
6	β	0.01	0.056	0.154	0.273	1
6	P	-24.65	-0.046	0.136	0.998	62.843

Table S1. Estimated SFL parameters in Experiments 1-6.

Section 1: Computational Models

To quantify how well the SFL model fits our experimental data, we compared it to a range of different models that previously have been used to characterise social learning strategies, as well as variations of these models (cf. Figure 5 of the main text). Below, we detail the formulations of these models.

S1.1 Fixed Heuristics. The basic *Fixed Heuristics*, also known as decision biasing[1], model is the standard computational model of the heuristic account of social learning strategies[1–4]. The model combines individual reward learning with a fixed social influence. It does not use features to estimate the value of candidate actions but instead learns the value of each action directly. Attractions (equivalent to Q-values) are updated through direct experience:

$$A(a)_{t+1} = (1 - \alpha)A(a)_t + \alpha R_t \quad [1]$$

where α is the individual learning rate, which determines the influence of the outcome on the updated attraction. The model uses a softmax function to compute the individual action probabilities:

$$P_I(a_i) = \frac{e^{A(a_i)/\beta}}{\sum_{j=1}^{k} e^{A(a_j)/\beta}} \quad [2]$$

Centrally, the individual action probabilities P_I are combined with social influence P_S using a weighting parameter to determine the total action probability:

$$P(a_i) = (1 - k)P_I(a_i) + kP_S(a_i) \quad [3]$$

where k ($0 \le k \le 1$) is a weighting parameter. The strength of the social influence depends on the ratio of social information for each option [2–5]:

$$P_S(a_i) = \frac{n_{i_t}^{d}}{\sum_{j=1}^{k} n_{j_t}^{d}} \quad [4]$$

where n_i is the number of others selecting option i (or , equivalently, others' payoff to action i). The strength of the social influence is regulated with a shape (or conformity) parameter d ($0 \le d$), which determines the strength of the majority or payoff bias. When $d = 1$, social learning is unbiased. As d becomes larger, the individual is more likely to select the majority/high payoff option. The fixed heuristics model has four free parameters: the learning rate α, the softmax temperature β, the social weight parameter k, and the shape parameter d. Crucially, the social influence is constant and not modified by experience.

S1.2 Value Shaping. In the "Value Shaping" model[5], others' choices or outcomes act as a pseudo-reward that directly shapes the observer's expectations (i.e., Q-values) about the value of different options. This allows social learning to adapt to the social environment. Specifically, the Q-values are updated by the observed social feature (proportion of others taking choice i or others' average payoff to choice i) before the individual's choice

$$Q(a_i)_t = Q(i)_t + \kappa_t \frac{n(a_i)_t}{\sum_{j=1}^{k} n(a_j)_t} \quad [5]$$

where κ_t ($0 \le \kappa_t \le 1$) is the social updating rate at time t, and $k = 2$. Choices follow a softmax function:

$$P(a_i) = \frac{e^{Q(a_i)/\beta}}{\sum_{j=1}^{k} e^{Q(a_j)/\beta}} \quad [6]$$

and learning a standard Q-learning update.

$$Q(a_i)_{t+1} = Q(a_i)_t + \alpha(R_t - Q(a_i)_t) \quad [7]$$

where α is the learning rate parameter ($0 \le \alpha \le 1$). Centrally, this model has a "meta-learning" mechanism, which dynamically modulates the social learning rate based on whether the demonstrator's actions ($Q(d)$) align with what the focal individual perceives as most valuable at a given time.

$$\kappa_{t+1} = \kappa_t + \alpha_s(\tau - \kappa_t) \quad [8]$$

$$\tau = \{1\ if\ \ Q(d) = \ max(Q_t)\}\ 0\ otherwise\ \} \quad [9]$$

In other words, the individual can learn whether the demonstrators' actions are aligned with their rewards, which, in principle, could allow it to fit our experimental design. While previous applications of the Value Shaping model assumed a reset of the social learning rate to 0 in new contexts[5], we relaxed this assumption to enable generalisation between the Learning and Test phases of our experiments. We tested two versions of the Value Shaping model, where the initial value of $\boldsymbol{\kappa}$ either was zero ("Value Shaping") or estimated as a free parameter ("Value Shaping + prior").

Section 2: Additional Analyses

S2.1 As per our Experiment 1 pre-registration, we conducted the following additional analysis: In addition to analysing learning effects on the first Test phase trial, we carried out analyses on the entire Test phase. Results of a logistic regression with the entire test phase closely mirror those which only focus on the first trial, β = -1.94, SE = 0.19, z = -9.96, p < .001.

S2.2 As per our Experiment 1 pre-registration, we also analysed the relationship between social information use and social network size. Participants responded to the Social Network Index (SNI) questionnaire[6]. We used logistic regression to test the effect of SNI on social information use on the first test phase trial. Analyses do not show an association, β = 0.049, SE = 0.03, t = 1.51, p = .13.

S2.3 Apart from predicting a difference between the Congruent and Incongruent conditions in Experiments 1-2, the SFL model also predicts that the difference should decrease across experimental trials in the Test phase. The reason is that the reward probability for both options

in the Test phase was 0.5, implying that the difference in feature weights between conditions should be gradually overlearned. This prediction held for both experiments, here tested by the interaction between Test phase trial and condition (Experiment 1: $\chi^2(1) = 17.5$, $p < .001$, Experiment 2: $\chi^2(1) = 2209.15$, $p < .001$).

S2.4 In Experiment 5, we found that individual differences in w_{Others} at the end of the learning phase predicted social influence in the BEAST task (Figure S4). To provide a model-independent counterpart to the SFL estimate, we also tested whether the proportion of majority choices that led to reward during the learning phase predicted S_{Beast}. This was the case: β = 0.077, SE = 0.028, t = 2.76, p = .006.

Moreover, we conducted an additional analysis to verify that the link between social feature weights and social influence scores (see Figure S4) was not driven by outliers. To accomplish this, we removed potential outlier data points with w_{Others}< -0.33 and reran the robust regression analysis. The result of this regression was similar to the original analysis: β = 0.21, SE = 0.09, t = 2.4, p = 0.017. We found a similar relationship with OLS regression (with possible outliers removed: β = 0.25, SE = 0.09, t = 2.85, p = .005. Including possible outliers: β = 0.21, SE = 0.7, t = 2.85, p = .005). We conclude that the relationship between the social feature weight and social influence is robust to outliers.

S2.5 As Experiment 6 involved two within-participant conditions, the order of these conditions might impact the results of the transfer phase. However, we did not find evidence that the estimated feature competition effect was influenced by condition order (Pair * Condition order: $\chi^2(1) = 0.14$, p = 0.29). In a second set of analyses, we tested whether individual variation in the number of rewards received in the two conditions was associated with the feature competition effect. Specifically, we added the absolute difference of average reward received in the two learning conditions as a predictor to the model. This analysis revealed no evidence

that reward differences accounted for the feature competition effect (Pair * Absolute reward difference: $\chi^2(1) = 0.68$, $p = 0.4$).

Section 3: Model Identifiability and Generalizability

S3.1 Model recovery. We conducted a model recovery analysis to test whether the model used to simulate the data would also provide the best fit to that data. We based our analysis on Experiment 1, which serves as the basic test of the SFL model. To streamline the analysis, we focused on the best-fitting models from the three model families of interest: SFL (both with and without the Prior parameter, as these models performed similarly), Fixed Heuristics, and Value Shaping (without Prior).

We first simulated data from these three models by randomly sampling parameter values (with replacement) from the set of estimated parameters. We generated 2000 synthetic participants from each model. The same fitting procedure used for the original data—individual-level maximum likelihood estimation followed by Bayesian random-effects model comparison—was applied to these simulated datasets.

First, we constructed a standard confusion matrix (Fig. S14a). This matrix shows the "probability that data generated by one model is best fit by another" [7]. The results indicate perfect recovery for the SFL model (here first without a Prior parameter). However, the SFL model also fits data generated by the Fixed Heuristics and Value Shaping models reasonably well. One interpretation of the fact that the SFL model fits all simulations reasonably well is that its parsimony and flexibility allow it to assume the behavior of a range of models.

Although the confusion matrix indicates perfect recovery concerning the SFL model, the recovery of the Fixed Heuristics and Value Shaping models was relatively poor. In

response, we conducted a second step: We created the "inversion matrix"[7], which addresses the more intuitive question: "Given that model B fits the data best, which model is most likely to have generated the data?" This metric is particularly relevant when the true generating model is unknown, as in empirical applications. The inversion matrix is generated by normalizing the columns of the confusion matrix[7].

The inversion matrix (Fig. S14b) demonstrates that values are maximised on the diagonal, suggesting that each model is most likely to have generated itself (conditional on fitting the data best). However, the SFL model only shows an inversion probability of ~0.5, suggesting some mimicry among the models. To assess the reliability of this probability, we computed bootstrap confidence intervals of the inversion matrix (calculated based on 1000 samples of 250 participants each). This analysis consistently yields inversion probabilities for the SFL model around ~0.5, and the 95% CI [0.46, 0.51] did not overlap with chance (~0.34), indicating that the SFL inversion probability is reliable.

For the SFL model with Prior, the confusion and inversion probabilities (see Fig. S15) are slightly lower, likely because the additional Prior parameter allows this variant to fit more extreme patterns that the other models may generate. Nonetheless, bootstrap confidence intervals of the inversion matrix demonstrate the inversion probability of the SFL+prior model is reliably higher than chance (bootstrap mean = 0.41, 95% CI [0.39, 0.44], vs. chance P = 0.34), again indicating that this difference is reliable.

S3.2 Generalizability. To evaluate the validity of the SFL model beyond those model recovery analyses, we tested its generalisability by generating out-of-sample predictions. Generalization outside the original experimental context is a robust, gold-standard measure of model validity[7]. Our approach follows the generalization criterion method[8]: Based on the mean and median parameters estimated from Experiment 1, we calculated, per participant and

model, the out-of-sample log-likelihood for Experiments 2-5. The log-likelihood was used as model evidence for the Bayesian model comparison procedure. This approach does not entail any additional model fitting for Experiments 2-5; a benefit of which is that overfitted models will perform worse, as, by definition, such models will only fit the training sample (Experiment 1) well. Hence, if the SFL model overfit in-sample data and therefore mimics other models, this would result in poor out-of-sample predictions. On the contrary, analyses show that the SFL demonstrates successful out-of-sample performance (Fig. 5b, Fig. S6, & Fig. S7), highlighting the SFL model's ability to generalize across different situations. This indicates that the SFL model did not overfit the in-sample data.

Although a comparison between the SFL and Value Shaping models based on median parameter estimates from Experiment 1 (Fig. S6) shows comparable out-of-sample predictions for Experiments 4 & 6, a comparison based on the mean estimated parameters clearly favored the SFL model (Fig. S7). Together, these results provide strong evidence that the SFL model offers the best generalization performance across experiments.

Section 4: Additional pre-registered analyses

S4.1. Exclusion Criteria. Our Experiment 1 pre-registration specifies that we would exclude participants who failed to select the high-reward option above chance during the Learning phase. Repeating the main analysis, where we test the effect of condition on following the majority on the first Test phase trial, with this exclusion criterion, shows similar effects to not applying the criterion: $\beta = -3.27$, $SE = 0.48$, $t = -6.85$, $p < .001$

S4.2. Terminology. In our Experiment 2 pre-registration, we refer to the Test phase as 'Transfer test'. To reduce ambiguity, we have opted to reserve the 'Transfer' descriptor for the Experiment 6 Transfer phase.

S4.3. Feature competition predictions. Although our pre-registration for Experiment 6 specifies that learning will be impaired for targets presented alongside social cues compared to those presented without social cues, it did not specify that a comparison of the relevant pairs (high-value pairs from both conditions and low-value pairs from both conditions) is required to test the central prediction.

Supplementary References

1. Najar, A., Bonnet, E., Bahrami, B. & Palminteri, S. The actions of others act as a pseudo-reward to drive imitation in the context of social reinforcement learning. *PLoS Biol.* **18**, e3001028 (2020).
2. McElreath, R. *et al.* Beyond existence and aiming outside the laboratory: estimating frequency-dependent and pay-off-biased social learning strategies. *Philos. Trans. R. Soc. Lond. B Biol. Sci.* **363**, 3515–3528 (2008).
3. Deffner, D., Kleinow, V. & McElreath, R. Dynamic social learning in temporally and spatially variable environments. *R. Soc. Open Sci.* **7**, (2020).
4. Efferson, C., Lalive, R., Richerson, P. J., McElreath, R. & Lubell, M. Conformists and mavericks: the empirics of frequency-dependent cultural transmission. *Evol. Hum. Behav.* **29**, 56–64 (2008).
5. McElreath, R. *et al.* Applying evolutionary models to the laboratory study of social learning. *Evol. Hum. Behav.* **26**, 483–508 (2005).
6. Cohen, S., Doyle, W. J., Skoner, D. P., Rabin, B. S. & Gwaltney, J. M., Jr. Social ties and susceptibility to the common cold. in *Foundations in Social Neuroscience* 1269–1278 (The MIT Press, 2002).
7. Wilson, R. & Collins, A. Ten simple rules for the computational modeling of behavioral data. *eLife* (2019) doi:10.31234/OSF.IO/46MBN.
8. Busemeyer, J. R. & Wang, Y. M. Model comparisons and model selections based on generalization criterion methodology. *J. Math. Psychol.* **44**, 171–189 (2000).
