# Supplementary material for: Feature-based reward learning shapes human social learning strategies
Source: Nat Hum Behav. 2025 Jul 23;9(10):2183–98. doi: 10.1038/s41562-025-02269-4 (PMC12545201; doi:10.1038/s41562-025-02269-4)
Supplement: Supplementary file 2 — Reporting Summary [file 41562_2025_2269_MOESM2_ESM.pdf]

nature portfolio

Corresponding author(s): Björn Lindström

Last updated by author(s): Apr 26, 2025

Reporting Summary

Nature Portfolio wishes to improve the reproducibility of the work that we publish. This form provides structure for consistency and transparency in reporting. For further information on Nature Portfolio policies, see our Editorial Policies and the Editorial Policy Checklist.

Statistics

For all statistical analyses, confirm that the following items are present in the figure legend, table legend, main text, or Methods section.

n/a	Confirmed	
☐	☒	The exact sample size (*n*) for each experimental group/condition, given as a discrete number and unit of measurement
☐	☒	A statement on whether measurements were taken from distinct samples or whether the same sample was measured repeatedly
☐	☒	The statistical test(s) used AND whether they are one- or two-sided<br>*Only common tests should be described solely by name; describe more complex techniques in the Methods section.*
☐	☒	A description of all covariates tested
☐	☒	A description of any assumptions or corrections, such as tests of normality and adjustment for multiple comparisons
☐	☒	A full description of the statistical parameters including central tendency (e.g. means) or other basic estimates (e.g. regression coefficient) AND variation (e.g. standard deviation) or associated estimates of uncertainty (e.g. confidence intervals)
☐	☒	For null hypothesis testing, the test statistic (e.g. *F*, *t*, *r*) with confidence intervals, effect sizes, degrees of freedom and *P* value noted<br>*Give P values as exact values whenever suitable.*
☒	☐	For Bayesian analysis, information on the choice of priors and Markov chain Monte Carlo settings
☐	☒	For hierarchical and complex designs, identification of the appropriate level for tests and full reporting of outcomes
☐	☒	Estimates of effect sizes (e.g. Cohen's *d*, Pearson's *r*), indicating how they were calculated

Our web collection on statistics for biologists contains articles on many of the points above.

Software and code

Policy information about availability of computer code

Data collection	The tasks were administered online and were programmed using the platform psiTurk (version 3.3.1)
Data analysis	We used R (version 4.3.0) and the lme4 package (version 1.1-35) to analyze the data. We used the MASS package (version 7.3-65) for robust regression.

For manuscripts utilizing custom algorithms or software that are central to the research but not yet described in published literature, software must be made available to editors and reviewers. We strongly encourage code deposition in a community repository (e.g. GitHub). See the Nature Portfolio guidelines for submitting code & software for further information.

Data

Policy information about availability of data

All manuscripts must include a data availability statement. This statement should provide the following information, where applicable:

- Accession codes, unique identifiers, or web links for publicly available datasets
- A description of any restrictions on data availability
- For clinical datasets or third party data, please ensure that the statement adheres to our policy

The data are available at the Open Science Framework: https://osf.io/jry9x/

Research involving human participants, their data, or biological material

Policy information about studies with human participants or human data. See also policy information about sex, gender (identity/presentation), and sexual orientation and race, ethnicity and racism.

Reporting on sex and gender	Participants self-reported gender. We did not perform any gender-based analysis, since our theoretical model did not make any predictions concerning gender-differences. Furthermore, reporting gender was not mandatory during participation, meaning that not all participants provided this data.
Reporting on race, ethnicity, or other socially relevant groupings	No reporting is made of race, ethnicity, or other socially relevant grouping variables. Our theoretical model made no apriori predictions about such variables.
Population characteristics	See above.
Recruitment	The participants were recruited on Prolific Academic. The study was described as a "decision-making experiment". Given this, we belive it is unlikely that self-seletion or related biases impacted the results.
Ethics oversight	Karolinska Institutet

Note that full information on the approval of the study protocol must also be provided in the manuscript.

Field-specific reporting

Please select the one below that is the best fit for your research. If you are not sure, read the appropriate sections before making your selection.

☐ Life sciences ☒ Behavioural & social sciences ☐ Ecological, evolutionary & environmental sciences

For a reference copy of the document with all sections, see nature.com/documents/nr-reporting-summary-flat.pdf

Behavioural & social sciences study design

All studies must disclose on these points even when the disclosure is negative.

Study description	In the basic experiment, participants learned which of two options (colored squares) yielded rewards through repeated choices. These two choice options were accompanied by social information, such as the ostensible choices of 100 previous participants, which were either positively or negatively associated with a participant's own reward. In this way, participants could learn to associate social information with own reward. In a subsequent test phase, participants faced two novel choice options, again presented alongside social information. We tested whether the learning phase shaped the test phase, such that participants were expected to be more likely to copy the majority if they had learned that majorities predict rewards, and more likely to copy the minority if they had learned that minorities predict rewards. Subsequent experiments varied this basic experimental design.
Research sample	The research sample consisted of participants recruited on Prolific Academic. Participants were required to be fluent in English, but not required to be native speakers. Their mean age was 33.8 years (sd = 11.96). All participants provided informed consent before starting the Experiment. Among participants who provided gender information, 931 identified as female, 957 as male, and 21 as other.<br>The sample was not representative. Because the purpose of the study was to test a theoretical model that in its current form does not make any predictions about participant characteristics, this sample was deemed appropriate.
Sampling strategy	We recruited participants who fulfilled the requirements outlined above at random from Prolific. Simulation-based power analysis was conducted for Experiments 1. The SFL model was simulated under random parameter values for a large number of repetitions . We assessed the % of runs where our planned analysis method provided a statistically significant result, given a true effect. For study 1, this indicated that 150 participants per group provided a power exceeding 0.9 of detecting a difference between the groups in the first Test phase trial. No power analysis was conducted for the following experiments but their sample sizes were chosen to be similar to Experiment 1
Data collection	The tasks were administered online and were programmed using HTML, CSS and JavaScript within the platform psiTurk. Prolific Academic was used for recruitment. Conditions were automatically randomized without the researchers' involvement.
Timing	For Experiment 1, we started and stopped collecting data in October 2022. For Experiments 2 & 6, we started collecting data in March 2023 and stopped collecting data in April 2023. For Experiment 5, we started and stopped collecting data in June 2023. For Experiments 3 & 4, we started collecting data in September 2024 and stopped collecting data in October 2024.
Data exclusions	No data were excluded.
Non-participation	No participants dropped out.
Randomization	Allocation to experimental groups was random, and controlled by the experimental software.

Reporting for specific materials, systems and methods

We require information from authors about some types of materials, experimental systems and methods used in many studies. Here, indicate whether each material, system or method listed is relevant to your study. If you are not sure if a list item applies to your research, read the appropriate section before selecting a response.

Materials & experimental systems

n/a	Involved in the study	
☒	☐	Antibodies
☒	☐	Eukaryotic cell lines
☒	☐	Palaeontology and archaeology
☒	☐	Animals and other organisms
☒	☐	Clinical data
☒	☐	Dual use research of concern
☒	☐	Plants

Methods

n/a	Involved in the study	
☒	☐	ChIP-seq
☒	☐	Flow cytometry
☒	☐	MRI-based neuroimaging

Plants

Seed stocks	*Report on the source of all seed stocks or other plant material used. If applicable, state the seed stock centre and catalogue number. If plant specimens were collected from the field, describe the collection location, date and sampling procedures.*
Novel plant genotypes	*Describe the methods by which all novel plant genotypes were produced. This includes those generated by transgenic approaches, gene editing, chemical/radiation-based mutagenesis and hybridization. For transgenic lines, describe the transformation method, the number of independent lines analyzed and the generation upon which experiments were performed. For gene-edited lines, describe the editor used, the endogenous sequence targeted for editing, the targeting guide RNA sequence (if applicable) and how the editor was applied.*
Authentication	*Describe any authentication procedures for each seed stock used or novel genotype generated. Describe any experiments used to assess the effect of a mutation and, where applicable, how potential secondary effects (e.g. second site T-DNA insertions, mosiacism, off-target gene editing) were examined.*
